# Resistance training in humans and mechanical overload in rodents do not elevate muscle protein lactylation

**DOI:** 10.3389/fphys.2023.1281702

**Published:** 2023-09-28

**Authors:** Madison L. Mattingly, Bradley A. Ruple, Casey L. Sexton, Joshua S. Godwin, Mason C. McIntosh, Morgan A. Smith, Daniel L. Plotkin, J. Max Michel, Derick A. Anglin, Nicholas J. Kontos, Shengyi Fei, Stuart M. Phillips, C. Brooks Mobley, Ivan Vechetti, Christopher G. Vann, Michael D. Roberts

**Affiliations:** ^1^ School of Kinesiology, Auburn University, Auburn, AL, United States; ^2^ Department of Physiology and Aging, University of Florida, Gainesville, FL, United States; ^3^ Department of Genetics, Standford University, Stanford, CA, United States; ^4^ Department of Nutrition and Health Sciences, University of Nebraska-Lincoln, Lincoln, NE, United States; ^5^ Department of Kinesiology, McMaster University, Hamilton, ON, Canada; ^6^ Duke Molecular Physiology Institute, Duke University School of Medicine, Duke University, Durham, NC, United States

**Keywords:** skeletal muscle, hypertrophy, lactate, lactylation, posttranslational modification

## Abstract

Although several reports have hypothesized that exercise may increase skeletal muscle protein lactylation, empirical evidence in humans is lacking. Thus, we adopted a multi-faceted approach to examine if acute and subchronic resistance training (RT) altered skeletal muscle protein lactylation levels. In mice, we also sought to examine if surgical ablation-induced plantaris hypertrophy coincided with increases in muscle protein lactylation. To examine acute responses, participants’ blood lactate concentrations were assessed before, during, and after eight sets of an exhaustive lower body RT bout (n = 10 trained college-aged men). Vastus lateralis biopsies were also taken before, 3-h post, and 6-h post-exercise to assess muscle protein lactylation. To identify training responses, another cohort of trained college-aged men (n = 14) partook in 6 weeks of lower-body RT (3x/week) and biopsies were obtained before and following the intervention. Five-month-old C57BL/6 mice were subjected to 10 days of plantaris overload (OV, n = 8) or served as age-matched sham surgery controls (Sham, n = 8). Although acute resistance training significantly increased blood lactate responses ∼7.2-fold (*p* < 0.001), cytoplasmic and nuclear protein lactylation levels were not significantly altered at the post-exercise time points, and no putative lactylation-dependent mRNA was altered following exercise. Six weeks of RT did not alter cytoplasmic protein lactylation (*p* = 0.800) despite significantly increasing VL muscle size (+3.5%, *p* = 0.037), and again, no putative lactylation-dependent mRNA was significantly affected by training. Plantaris muscles were larger in OV *versus* Sham mice (+43.7%, *p* < 0.001). However, cytoplasmic protein lactylation was similar between groups (*p* = 0.369), and nuclear protein lactylation was significantly lower in OV *versus* Sham mice (*p* < 0.001). The current null findings, along with other recent null findings in the literature, challenge the thesis that lactate has an appreciable role in promoting skeletal muscle hypertrophy.

## Introduction

Skeletal muscle possesses remarkable plasticity, allowing it to adapt and undergo structural and functional changes in response to various stimuli including exercise training (Hoppeler, 2016; Egan and Sharples, 2023; Roberts et al., 2023). Adaptations to exercise are regulated by intricate molecular mechanisms, including epigenetic modifications that influence gene expression (McGee and Hargreaves, 2019). Common epigenetic modifications include histone acetylation and DNA methylation, and both phenomena play roles in regulating gene expression through altering transcriptional accessibility. While epigenetic modifications have been investigated in several scientific disciplines, they have been implicated in the skeletal muscle anabolic responses following acute and chronic resistance exercise training (Pillon et al., 2020; Egan and Sharples, 2023; Sharples and Turner, 2023).

Lactate is a metabolite that is traditionally viewed as a metabolic byproduct, albeit it has recently been reported to serve as a substrate for histone lactylation. Zhang et al. (2019) recently established a novel function whereby rising lactate concentrations in cells treated with pro-inflammatory ligands altered histones via lysine lactylation. Through a series of validation experiments, these researchers reported that histone lysine lactylation is the product of a lactyl group being added to lysine residues of histones. Specifically, it was discovered that elevated lactate concentrations in cells could promote the expression of histone lactylation-specific genes (Zhang et al., 2019). Since this discovery, protein lactylation has been heavily investigated, but primarily in the context of cancer biology (Jiang et al., 2021; Lv et al., 2023). Lactate, while being a byproduct of glycolysis during higher intensity exercise, can also serve as important fuel source and gluconeogenic precursor (Rogatzki et al., 2015; Brooks, 2018). In this regard, blood lactate concentrations can range from 1–2 mmol/L at rest, exceed concentrations of 20 mmol/L during vigorous exercise (Withers et al., 1991; Goodwin et al., 2007), and intramuscular concentrations during exercise are likely much higher (MacDougall et al., 1999).

Lactate’s influence on epigenetic modifications in skeletal muscle adaptation to exercise has been mentioned (Seaborne and Sharples, 2020), but there has only been one investigation to our knowledge examined intricate interplay between exercise-induced increases in blood lactate and skeletal muscle protein lactylation changes (Huang et al., 2023). Given that this knowledge gap presents an intriguing opportunity, we sought to investigate if: 1) transient increases in blood lactate during and following a bout of exhaustive resistance exercise affects skeletal muscle protein lactylation; specifically proteins in both the nuclear and cytoplasmic fractions, 2) histone lactylation-specific genes are transiently altered following a bout of exhaustive resistance exercise, 3) long-term resistance training, which resulted in skeletal muscle hypertrophy, promotes alterations in skeletal muscle protein lactylation and/or the alteration in histone lactylation-specific genes, and 4) five-month-old mice subjected to 10 days of plantaris overload presented alterations in skeletal muscle protein lactylation. While the data in this area are limited data, we hypothesized that each mode of mechanical overload would increase skeletal muscle protein lactylation markers.

## Methods

### Ethical approval for human and rodent work

Human muscle obtained from a previously published studies by Sexton et al. (Sexton et al., 2023) and Vann et al. (Vann et al., 2022) was used for the acute and 6-week studies, respectively (IRB approval #: 20-081MR 2003 and IRB approval #: 19-245MR 1907). Inclusion criteria for both studies: i) 18-35 years old, ii) BMI <35 kg/m^2^ iii) free of cardio-metabolic diseases or any conditions that preclude the collection of a skeletal muscle biopsy, and iv) a self-reported resistance training >1 year at least three times weekly, and v) a tested barbell back squat of ≥1.5× bodyweight [estimated from a 3-repetition maximum (3RM) test]. All experimental procedures involving mice were approved by the University of Nebraska Institutional Animal Care and Use Committee (approval #: 2162).

### Study designs

**FIGURE 1:**
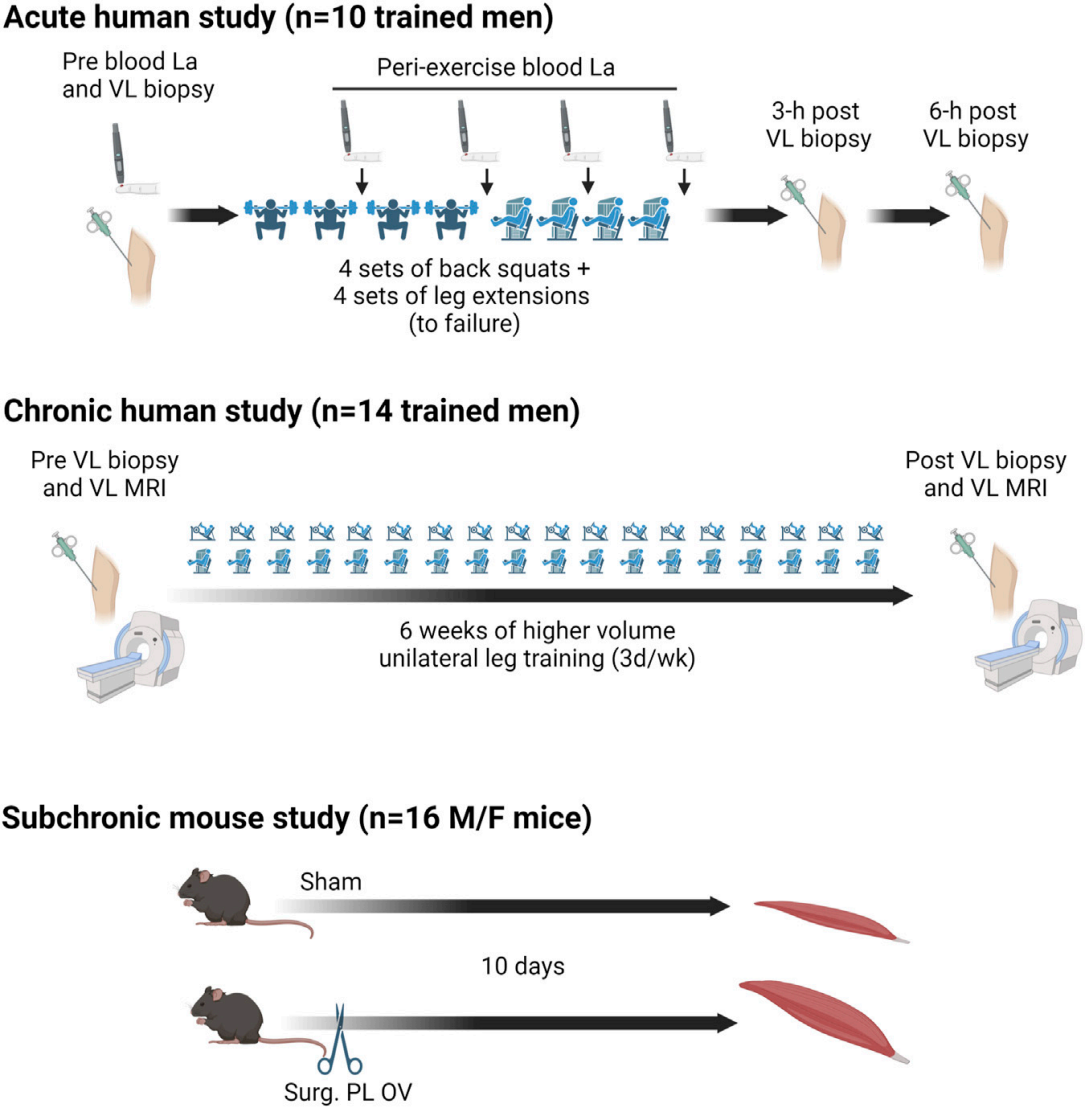
Study designs **Legend**: This illustration provides details about the acute human study, 6-week human training study, and rodent study. More experimental details are provided in-text.

The study designs for all aims are presented in Figure 1. For the acute human aim, participants completed a resistance exercise bout in the morning (between 7:00 a.m.–10:00 a.m.) following an overnight fast. The muscle analyzed herein was from the “30 Fail” bout whereby participants performed four sets of back squats and four sets of leg extensions at 30% of their estimated one-repetition maximum until volitional exhaustion. Capillary blood was obtained via finger sticks at five time points including before exercise, after the second set of squats, after the fourth set of squats, after the second set of leg extensions, and after the final set of leg extensions. Blood lactate concentrations were analyzed in real-time using a handheld lactate analyzer (Lactate Plus, Nova Biomedical, Mississauga, Ontario, Canada). Participants reporting nausea, malaise, or light-headedness were provided a cereal bar (calories: 170, total fat: 8 g, carbohydrates: 20 g, protein: 4 g) and a sports drink (calories: 80, carbohydrates: 21 g) to reduce symptoms. After the exercise bout, participants were instructed to return to the laboratory 3 and 6 h later for post-exercise biopsies. Participants were also instructed not to eat or perform activities beyond standing and walking during this time frame. More details on this study can be found in Sexton et al. (Sexton et al., 2023).

For the 6-week human study, participants performed progressive unilateral lower-body resistance training 3 days per week in conjunction with compound upper body exercises (i.e., barbell bench press, pronated grip barbell row, barbell stiff-leg deadlift). Participants’ legs were randomly assigned to lower-body training conditions before the start of the study, where some participants performed higher volume (HV) training on the left leg and higher load (HL) training on the right leg, or *vice versa*. Pre- and post-intervention testing sessions, with the latter being 72 h following the last training bout, involved vastus lateralis (VL) biopsies and mid-thigh magnetic resonance imaging (MRI) for VL muscle cross sectional area assessments. For the current analysis, only the pre- and post-intervention HV leg biopsies and MRI data (for VL muscle cross-sectional area) were analyzed. The HV training involved participants performing only the single-leg leg press and single-leg leg extension exercises. Training load and volumes were as follows: week 1, 5 sets of 10 repetitions per exercise at ∼60% 1RM; week 2, 6 sets of 10 repetitions per exercise at ∼60% 1RM; week 3, 7 sets of 10 repetitions per exercise at ∼60% 1RM; week 4, 8 sets of 10 repetitions per exercise at ∼60% 1RM; week 5, 9 sets of 10 repetitions per exercise at ∼60% 1RM; week 6, 10 sets of 10 repetitions per exercise at ∼60% 1RM. More details on training as well as pre- and post-intervention testing sessions are described in a previously published paper from our laboratory (Vann et al., 2022).

In the subchronic mouse aim, bilateral synergist ablation surgeries were performed as previously described (Vechetti et al., 2019) to induce plantaris muscle hypertrophy in 5-mo old C57BL/6 mice (OV, n = 4 males and 4 females). Briefly, mice undergoing OV were anesthetized (3% isoflurane with 1.5 L of O_2_ per minute) and placed in sternal recumbence and a small incision was made on the dorsal aspect of the lower hind limb with approximately half of the gastrocnemius and entire soleus muscle carefully excised. Age-matched control mice (Sham, n = 4 males and 4 females) were similarly placed in sternal recumbence, and a small incision was made on the dorsal aspect of the lower hind limbs without the removal of musculature. Ten days following OV and Sham surgeries, mice were euthanized via CO_2_ inhalation followed by cervical dislocation. Plantaris muscles were immediately excised, weighed, snap frozen in liquid nitrogen, and stored at −80°C, and shipped to Auburn University for analysis. During experimentation, all mice were housed in a temperature and humidity-controlled room and maintained on a 14:10-h light-dark cycle with food and water *ad libitum*.

#### Wet laboratory analyses

*Western blotting.* In the acute human study, cytoplasmic and nuclear lysates were obtained as previously described by Sexton et al. (Sexton et al., 2023). In the human training study, cytoplasmic lysates were obtained as previously described by Vann et al. (Vann et al., 2022). Lysates were batch process-assayed for total protein content using a BCA Protein Assay Kit (Thermo Fisher; Waltham, MA, United States) and prepared for Western blotting using 4x Laemmli buffer at 1 μg/μL. Thereafter, 15 μL of prepped samples were loaded onto 4%–15% SDS-polyacrylamide gels (Bio-Rad; Hercules, CA, United States) and subjected to electrophoresis (180 V for 50 min) using pre-made 1x SDS-PAGE running buffer (VWR; Radnor, PA, United States). Proteins were then transferred (200 mA for 2 h) to polyvinylidene difluoride membranes (Bio-Rad), Ponceau stained and imaged to ensure equal protein loading between lanes. Membranes were then blocked for 1 h at room temperature with 5% nonfat milk powder in Tris-buffered saline with 0.1% Tween-20 (TBST; VWR). Membranes were then rocked for 72 h at 4°C with an anti-lactyl lysine antibody (PTM-1401; PTM Bio Inc., Chicago, IL, United States) at a 1:2000 dilution in TBST with 5% bovine serum albumin (BSA). Membranes were subsequently incubated with horseradish peroxidase-conjugated anti-rabbit antibodies (1:2000; Cell Signaling) in TBST with 5% BSA at room temperature for 1 h. Membrane development was performed using an enhanced chemiluminescent reagent (Luminata Forte HRP substrate; Millipore Sigma), and band densitometry was performed using a gel documentation system and associated densitometry software (ChemiDoc Touch, Bio-Rad). Densitometry values of protein targets were corrected for Ponceau densities and normalized to Pre values for each study (human samples) or Sham animals (mouse samples).

*Data mining for mRNAs of interest in the human aims.* Genes of interest were queried based on the initial report by Zhang et al. (2019), which indicated the histone lactylation affects the expression of multiple mRNAs in bacterially challenged M1 macrophages. According to Figure 3 (panel h) in their paper, H3K18la-specific genes included ARG1, MMP9, RTN4R, TGM1, SPSB4, and HSD11B1. Since Zhang and colleagues also implied that p300-mediated H3 and H4 lactylation occurred, we also interrogated the mRNA for this gene (EP300). Finally, we interrogated the mRNA expression levels of MCT1/4 (SLC16A1 and SLC16A4, respectively) given that the associated transporter proteins are responsible for lactate uptake into myofibers (Kitaoka et al., 2022).

Transcriptome-wide mRNA analysis was performed for the acute human study as described by Sexton et al. (Sexton et al., 2023). Briefly, total RNA was isolated from ∼10 mg of skeletal muscle using Trizol (VWR; Radnor, PA, United States) according to manufacturer’s instructions. Following RNA isolation, purity was assessed using a NanoDrop Lite Spectrophotometer (Thermo Fisher, Waltham, MA, United States) and RNA was stored at −80°C until shipment on dry ice to a commercial laboratory for transcriptomic analysis using the Clariom S Assay Human mRNA array (North American Genomics; Decatur, GA, United States). Raw data were received as.CEL files, mRNA expression data were extracted using the Transcriptome Analysis Console v4.0.2 (Thermo Scientific), and mRNAs of interest were manually extracted for statistical analysis.

In the human 6-week training study, total RNA was isolated from ∼10 mg of skeletal muscle using Ribozol (Ameresco; Framingham, MA, United States) following the manufacturer’s specifications. RNA purity was assessed using a NanoDrop Lite Spectrophotometer (Thermo Fisher, Waltham, MA, United States), and RNA was then stored at −80 ° C until shipment to McMaster University for analysis. Total RNA (∼500 ng) underwent two rounds of amplification using a commercially available kit (Affymetrix, Santa Clara, CA, United States). For analysis, 30 μg of biotinylated cRNA from each sample was then hybridized to the Affymetrix Human Transcriptome Array (HTA) 2.0 microarray. Resultant.CEL files were then read into R using the “oligo” and “affycoretools” package and subsequently annotated using the “hta20transcriptcluster.db” database. After the raw data was read and annotated, the robust multiarray algorithm (RMA) was used to normalize data, and mRNAs of interest were manually extracted for statistical analysis.

### Statistics

All statistical analyses were performed using jamovi (https://www.jamovi.org), and figures were constructed using paid site licenses for BioRender (https://www.biorender.com) and GraphPad Prism v9.2.0 (San Diego, CA, United States). For the acute human study, dependent samples t-tests were performed for dependent variables containing two points and one-way repeated measures ANOVAs with Tukey’s *post hoc* corrections were performed for dependent variables containing three time points. Pearson correlations were also performed on select variables. For the human training study and mouse data, dependent samples t-tests were performed for dependent variables. All data in-text and in figures are presented as mean ± standard deviation (SD) values, individual respondent data are also presented, and statistical significance was established as *p* < 0.05.

## Results

### Participant characteristics and training volume details

Participant characteristics for the acute study are presented by Sexton et al. (Sexton et al., 2023). Ten of the 11 participants from that dataset were examined in this study (23 ± 4 years old; 87.0 ± 12.4 kg) due to tissue limitations. Participant characteristics for the 6-week training study are presented by Vann et al. (Vann et al., 2022). Fourteen of the 15 previously trained college-aged men from that dataset were examined in this study (23 ± 3 years old; 89.0 ± 11.9 kg) due to tissue limitations.

Participants in the acute human study performed the following number of repetitions per squat set at 30% of their estimated 1-RM load (mean ± SD): set 1, 68 ± 14 repetitions; set 2, 41 ± 14 repetitions; set 3, 35 ± 12 repetitions; set 4, 29 ± 11 repetitions. Participants also performed the following number of repetitions per leg extension set at 30% of their estimated 1-RM load (mean ± SD): set 1, 25 ± 13 repetitions; set 2, 24 ± 8 repetitions; set 3, 25 ± 12 repetitions; set 4, 24 ± 8 repetitions. Hence, this totaled ∼280 repetitions for the bout.

Participants in the chronic training study performed 100 total repetitions of leg press and leg extensions during week 1 (50 repetitions per exercise), 120 total repetitions of leg press and leg extensions during week 2 (60 repetitions per exercise), 140 total repetitions of leg press and leg extensions during week 3 (70 repetitions per exercise), 160 total repetitions of leg press and leg extensions during week 4 (80 repetitions per exercise), 180 total repetitions of leg press and leg extensions during week 5 (90 repetitions per exercise), and 200 total repetitions of leg press and leg extensions during week 6 (100 repetitions per exercise). Notably, these repetitions occurred at ∼60% of the participants’ 1-RM.

### One bout of exhaustive resistance exercise increases blood lactate concentrations while minimally affecting muscle protein lactylation markers

**FIGURE 2:**
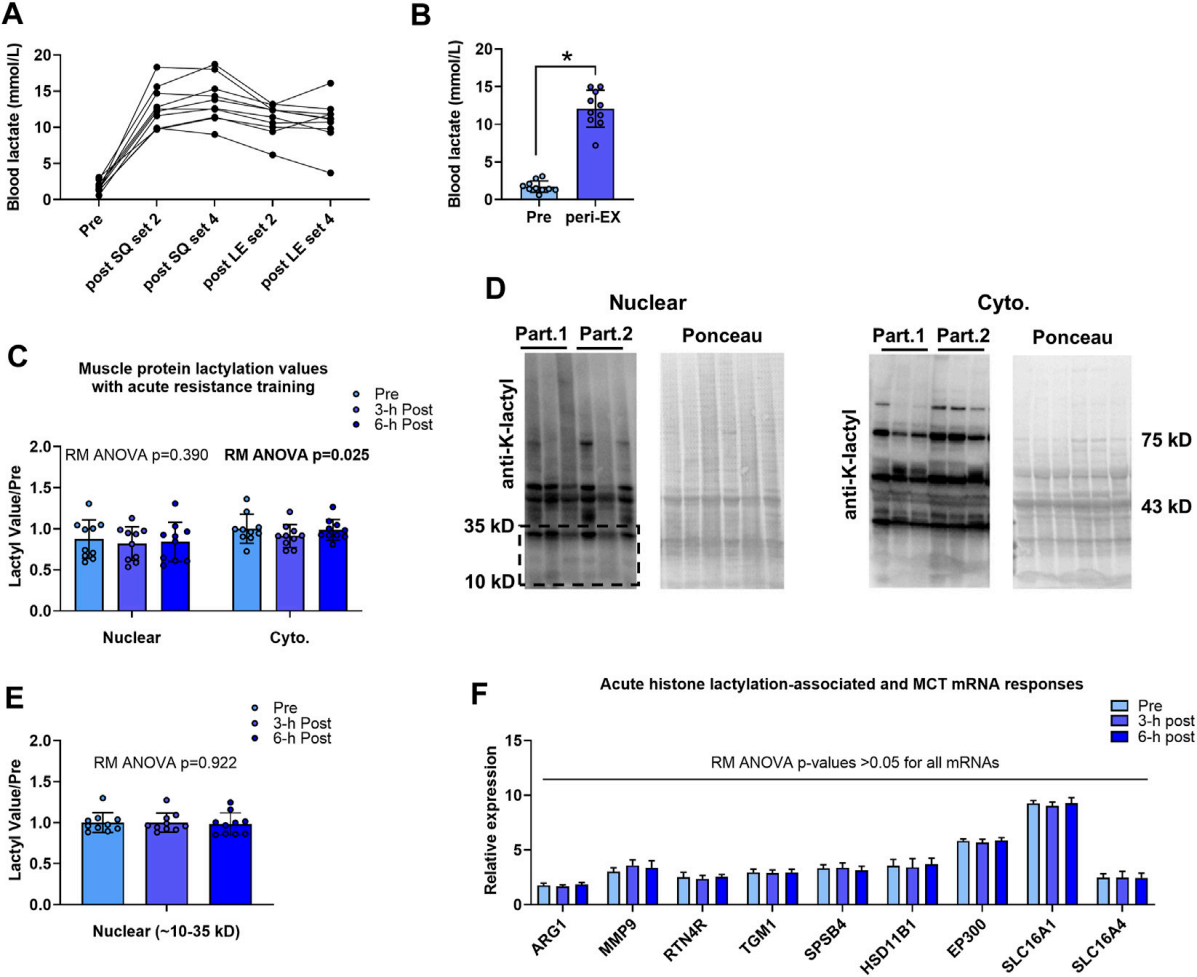
Acute bout human data **Legend**: Individual responses in blood lactate **(A)** and basal/pre-exercise relative to the average peri-exercise blood lactate concentrations indicate that a bout of exhaustive resistance exercise elicits a robust blood lactate response **(B)**. However, skeletal muscle cytoplasmic and nuclear protein lactylation levels remained unaltered 3-h and 6-h post-exercise **(C, E)**; representative Western blots are presented **(D)** whereby “Part. 1” and “Part. 2” signifies two representative human participants. Finally, putative histone lactylation-dependent mRNAs remained unaltered at post-exercise time points **(F)**. Symbol: *, peri-exercise > pre-exercise (*p* < 0.05).

Data associated with the acute bout effects on blood lactate concentrations and muscle protein lactylation markers are presented in Figure 2. Blood lactate levels were elevated in all participants during the resistance exercise bout (Figure 2A), and average peri-exercise blood lactate concentrations were significantly greater than resting levels (12.1 ± 0.8 mM *versus* 1.7 ± 0.8 mM, *p* < 0.001; Figure 2B). Although cytoplasmic protein lactylation demonstrated a significant model effect (repeated measures ANOVA *p* = 0.025), levels at 3-h and 6-h post-exercise were not significantly different compared to pre-exercise levels (*p* = 0.081 and *p* = 0.941, respectively; Figure 2C,D). Total nuclear protein lactylation was not significantly altered following the exercise bout (repeated measures ANOVA *p* = 0.390), and nuclear protein lactylation in the kilodalton region whereby histones are enriched (∼10–35 kD) was not significantly altered following the exercise bout (repeated measures ANOVA *p* = 0.922; Figure 2C,D,E). Finally, repeated measures ANOVA *p*-values for the H3K18la-specific genes, EP300, and SLC16A1/4 were all not statistically significant (*p* > 0.05).

### No significant associations existed between resistance exercise-induced increases in blood lactate concentrations and muscle protein lactylation markers

**FIGURE 3:**
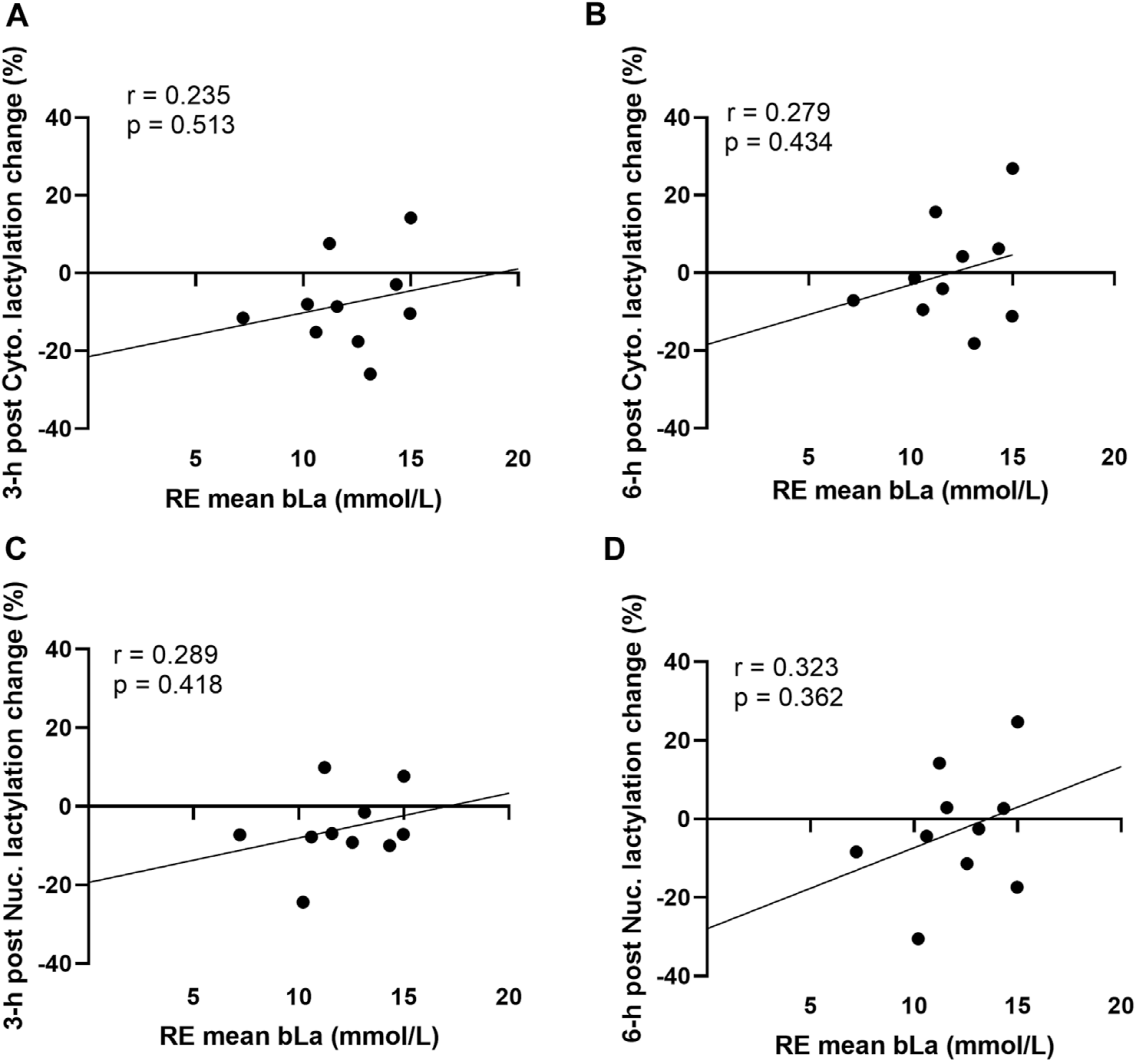
Acute bout human correlations **Legend**: These data demonstrate that no significant correlations existed between mean peri-exercise blood lactate concentrations and the percentage changes (from pre-exercise) in 3-h post-exercise cytoplasmic protein lactylation **(A)**, 6-h post-exercise cytoplasmic protein lactylation **(B)**, 3-h post-exercise nuclear protein lactylation **(C)**, and 6-h post-exercise nuclear protein lactylation **(D)**.

Acute resistance exercise bout correlations between peri-exercise blood lactate concentrations and muscle protein lactylation levels are presented in Figure 3. No significant associations were evident for peri-exercise blood lactate concentrations and percentage changes in cytoplasmic protein lactylation levels at 3-h and 6-h post-exercise (Figure 3A,B). Additionally, no significant associations were evident for peri-exercise blood lactate concentrations and percentage changes in nuclear protein lactylation levels at 3-h and 6-h post-exercise (Figure 3C,D).

### Six weeks of resistance training does not affect cytoplasmic protein lactylation despite promoting VL muscle hypertrophy

**FIGURE 4:**
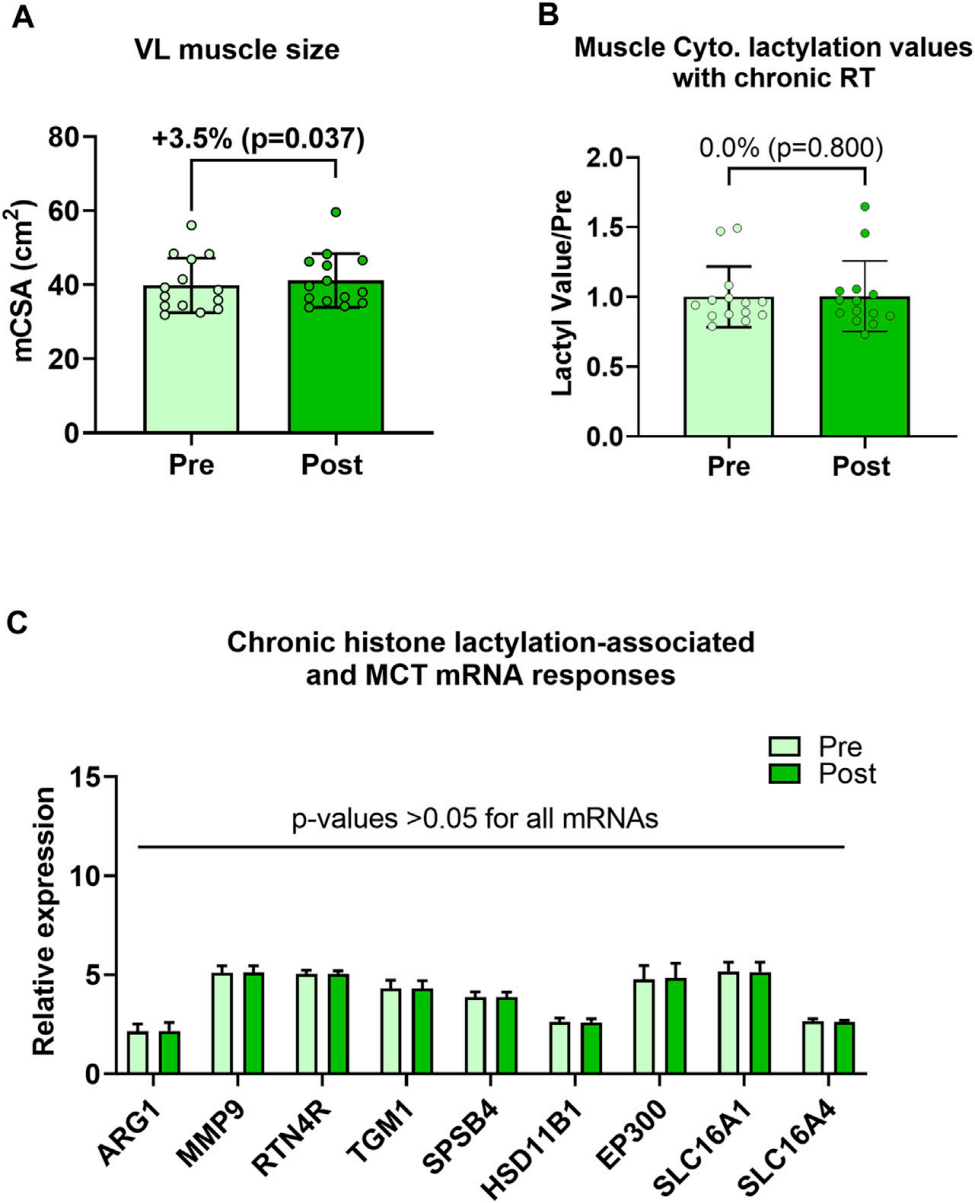
Six-week training outcomes in humans **Legend**: These data demonstrate that, while the 6-week training period increased vastus lateralis muscle cross-sectional area (mCSA) **(A)**, muscle cytoplasmic protein lactylation **(B)** or putative histone lactylation-dependent mRNAs **(C)** were not altered with training.

Human 6-week training study outcomes are presented in Figure 4. Although VL muscle size increased during the 6-week training period (39.9 ± 7.4 cm^2^ to 41.2 ± 7.4 cm^2^, *p* = 0.037; Figure 4A), cytoplasmic protein lactylation did not significantly change with training (*p* = 0.800; Figure 4B). Moreover, pre-to-post intervention *p*-values for H3K18la-specific genes, EP300, and SLC16A1/4 were all not statistically significant (*p* > 0.05; Figure 4C). Note that nuclear lysates were not available for these samples, and thus, nuclear protein lactylation was not able to be interrogated. Also note that the hypertrophy data in Figure 4A has been previously reported (Figure 4A in Vann et al. (Vann et al., 2022)); however, these data are included here for convenience to the reader.

### Ten days of functional overload induces plantaris hypertrophy in mice while not altering cytoplasmic protein lactylation and reducing nuclear protein lactylation

**FIGURE 5:**
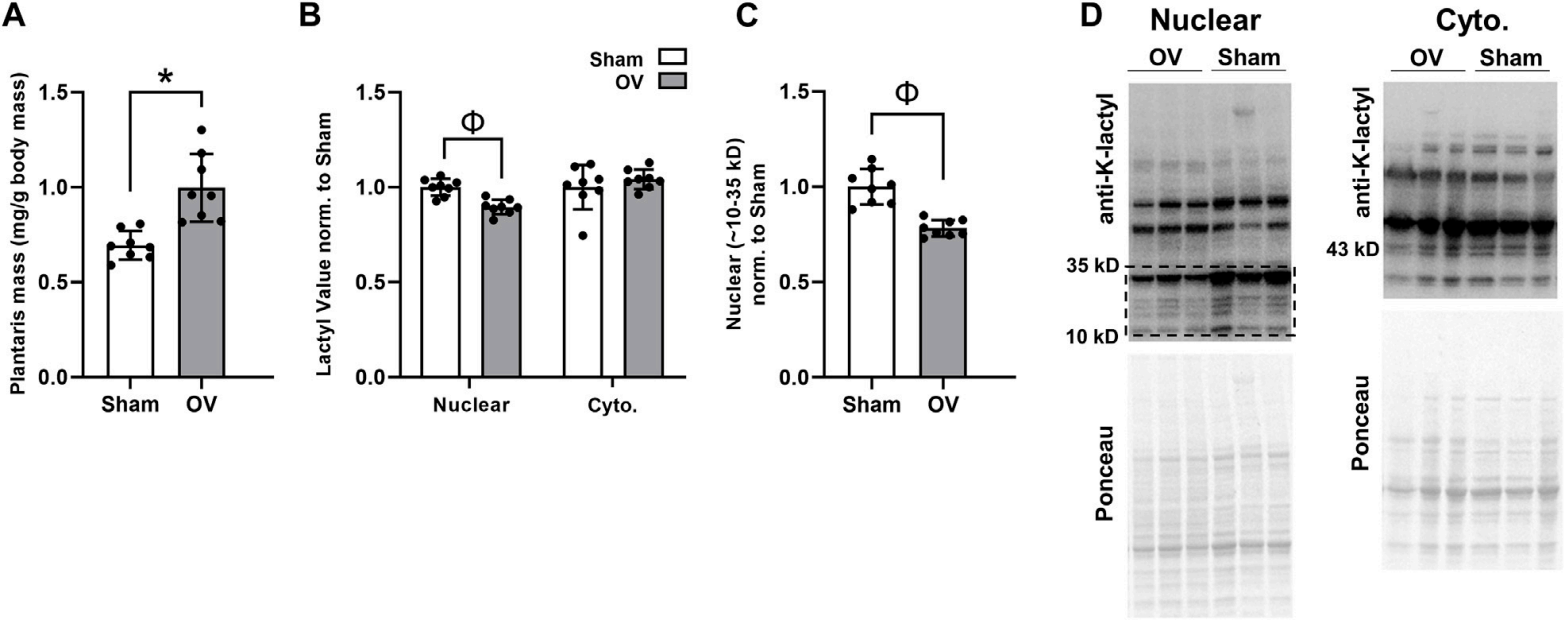
Ten-day overload outcomes in mice **Legend**: These data demonstrate that, while 10 days of functional overload in mice led to muscle hypertrophy relative to Sham mice **(A)**, muscle cytoplasmic protein lactylation levels were unaffected and nuclear protein levels were significantly lower **(B, C)**; representative Western blots are presented in panel **(D)**. Symbols: *, OV > Sham (*p* < 0.05); Φ, Sham > OV (*p* < 0.05).

Rodent outcomes are presented in Figure 5. Compared to Sham mice, OV plantaris masses (relative to body masses) were significantly greater following 10 days of overload (*p* < 0.001; Figure 5A). However, cytoplasmic protein lactylation levels were similar between cohorts (*p* = 0.369), and total nuclear protein lactylation levels (as well as nuclear protein lactylation levels between 10 and 35 kD) were significantly lower in OV *versus* Sham plantaris muscles (*p* < 0.001 for data in both Figure 5B,C). These trends were evident in both sexes; specifically, total nuclear protein lactylation levels were lower in OV *versus* Sham muscle in males (*p* = 0.005) and females (*p* = 0.041), and cytosolic protein lactylation levels were similar between muscles in males (*p* = 0.313) and females (*p* = 0.991).

## Discussion

The purpose of this study was to examine if markers of muscle protein lactylation increased with various modes of mechanical overload in humans and mice. Several findings went against our hypothesis. First, nuclear and cytoplasmic protein lactylation levels were not significantly altered following an exhaustive resistance exercise bout despite blood lactate concentrations being robustly affected. Additionally, 6 weeks of resistance training did not promote significant changes in cytoplasmic protein lactylation despite VL hypertrophy being observed. Also notable was that acute exercise or 6 weeks of resistance training did not affect histone lactylation-specific genes expression. Finally, 10 days of mechanical overload in mice did not affect cytoplasmic protein lactylation, and significantly decreased nuclear protein lactylation levels.

The recent 2019 report by Zhang et al. (2019) provided the first evidence that cellular proteins could be post-translationally modified via lysine lactylation. Specifically, the authors reported that 28 lactylation sites exist on core histones in human and mouse cells, hypoxic and bacterial challenges *in vitro* (which induce increases in lactate) stimulate histone lactylation, and a core set of histone lactylation-dependent mRNAs are modulated with these challenges. While these findings are provocative, sparse data exist connecting skeletal muscle histone or protein lactylation to exercise adaptations. Huang et al. (2023) recently reported that an increase in soleus muscle (as well as liver and adipose tissue) protein lactylation occurs 24 h following a bout of HIIT in rodents, and levels return to pre-exercise levels by 72 h post-exercise. While not in skeletal muscle, others have recently reported that exercise training in apolipoprotein-deficient mice promotes Mecp2 lysine lactylation (Mecp2k271la) in aortic tissue and that Mecp2k271la represses the expression of other genes through chromatin binding (Wang et al., 2023). Our study design differs from these rodent reports since we examined human and rodent skeletal muscle in response to mechanical overload. However, a current finding critical to note is that one bout of resistance exercise in humans upregulates blood (and muscle (MacDougall et al., 1999)) lactate concentrations despite not altering nuclear or cytoplasmic protein lactylation. Moreover, increased protein lactylation did not accompany skeletal muscle hypertrophy in humans or rodents. We interpret these data to suggest that protein lactylation, and/or the altered expression of histone lactylation genes, likely do not play an appreciable role in the adaptive responses to mechanical overload.

The decreased nuclear lactylation in the OV muscle mice is intriguing. One reason for this occurrence could be that, while not measured in the current study, others have reported that mechanical overload through synergist ablation does not alter blood lactate concentrations 1-12 days following surgery (Kitaoka et al., 2011). However, the authors from this study also reported that the mRNA expression of the MCT1/4 lactate transporters was significantly elevated one- and 3-days post-surgery, and protein levels were elevated 12 days post-surgery. Hence, it remains possible that synergist ablation increased the localized lactate production, and the upregulation in these transporters reflected an enhancement in lactate metabolism. Additionally, the reduction in nuclear protein lactylation observed in the current study could be due to overload-induced increases in protein acetylation. As stated, lysine lactylation is the product of a lactyl group being added to lysine residues of histones, and Zhang et al. (2019) experimentally demonstrated that histone lactylation competes with histone acetylation *in vitro*. Also notable are prior data from multiple studies demonstrating that synergist ablation increases nuclear protein acetylation (Hanna et al., 2009; von Walden et al., 2012). Hence, we speculate that the observed decrease in nuclear protein lactylation was likely due to nuclear proteins being acetylated at competitive lysine residues, and given that we lack data or additional lysates to confirm this hypothesis, this requires further investigation.

As an aside, there have been various hypotheses surrounding the potential role(s) lactate exerts as a signaling mediator during overload-induced skeletal muscle hypertrophy. For instance, a recent review by Lawson et al. (Lawson et al., 2022) discussed numerous studies reporting that mechanical overload-induced increases in blood lactate coincide with increased anabolic signaling, and that lactate may be a key signal for overload-induced hypertrophy. Moreover, some *in vitro* reports support lactate stimulating markers of myoblast activation (Willkomm et al., 2014; Oishi et al., 2015), which implies that lactate may increase satellite cell proliferation *in vivo*. However, there are conflicting data in this area. Shirai et al. (Shirai et al., 2022) reported that 14 days of lactate administration during synergist ablation-induced mechanical overload does not augment plantaris muscle hypertrophy in mice. These authors also reported that a single dose of lactate administration in a separate cohort of mice does not enhance the anabolic signaling or muscle protein synthesis responses to hindlimb stimulation, and similar human findings have been reported by others when administering lactate around and resistance exercise bout (Liegnell et al., 2020). Finally, it has been recently reported that obese individuals present higher levels of skeletal muscle protein lactylation (Maschari et al., 2022), and modest associations with glucose intolerance were also noted. Given our null findings along with these recent reports (Liegnell et al., 2020; Shirai et al., 2022), we posit that lactate has very little, if any, role in promoting or participating in skeletal muscle hypertrophy. Moreover, increased protein lactylation may be more indicative of metabolic perturbances than an exercise-specific adaptation, albeit this too needs to be further examined.

### Experimental considerations

Like many human studies with muscle biopsies, we examined a small number of participants with limited time points, and only trained men were studied herein. It should also be noted this is a secondary analysis whereby samples from previous in-house studies were utilized. Hence, time course studies examining similar outcomes in untrained populations and women will add further insight. Moreover, the mice utilized herein were naïve to overload. Hence, analyzing skeletal muscle from trained humans and overload-naïve mice is an unresolved limitation. Finally, two different microarray platforms were used for the human studies (Transcriptome Array (HTA) 2.0 microarray being used for the chronic study and the Clariom S Assay Human mRNA array being used for the acute study). While this likely did not affect outcomes within each study, there were certain targets (e.g., SLC16A1) showing relative expression differences between studies. Hence, this is an unresolved limitation.

## Conclusion

Acute resistance exercise resulting in increased blood lactate concentrations, and 6 weeks of resistance training which led to muscle hypertrophy, did not correspond with increases in any of the assayed protein lactylation markers. Moreover, extreme hypertrophy in rodents via synergist ablation decreases nuclear protein lactylation and does not alter cytoplasmic protein lactylation.

## Data Availability

The datasets presented in this study can be found in online repositories. The names of the repository/repositories and accession number(s) can be found below: https://www.ncbi.nlm.nih.gov/geo/, GSE220899.
